# Association between abdominal obesity and nutritional supplement use among Iranian adults in the Shahedieh cohort study

**DOI:** 10.1038/s41598-025-85136-6

**Published:** 2025-01-07

**Authors:** Sina Nouri, Akram Ghadiri-Anari, Saeed Hosseini, Narjes Hazar

**Affiliations:** 1 School of Medicine, Shahid Sadoughi University of Medical Sciences, Yazd, Iran; 2 Diabetes Research Center, Shahid Sadoughi University of Medical Sciences, Yazd, Iran; 3 Department of Epidemiology, School of Public Health, Iran University of Medical Sciences, Tehran, Iran; 4 Center for Healthcare Data Modeling, Department of Biostatistics and Epidemiology, School of Public Health, Shahid Sadoughi University of Medical Sciences, Yazd, Iran; 5 Diabetes Research Center, Shahid Sadoughi University of Medical Sciences, Yazd, Iran

**Keywords:** Obesity, Abdominal, Dietary supplements, Iran, Ferric Compounds, Epidemiology, Obesity

## Abstract

The relationship between the use of supplements and the presence of abdominal obesity is a topic that is currently being debated. The purpose of the present study was to evaluate the existence of such an association. The current cross-sectional investigation included a population of 9878 individuals who were the focus of the study’s initial phase in the Shahedieh cohort study, conducted in Yazd, Iran. Waist circumference was measured with the help of an inflexible tape measure. In addition, the data regarding the consumption of various supplements, including multivitamins, folic acid, ferric compounds, omega 3, calcium, vitamin D, zinc, and other supplements, were collected through interviews conducted by trained interviewers. Among the entire sample, 4785 individuals (50.6%) were found to have abdominal obesity, while 4093 individuals (43.3%) reported using at least one type of supplements. In the simple regression analysis, all of the supplements, except for multivitamins and zinc, were identified as adverse factors for abdominal obesity. However, in the multivariable regression analysis, only ferric compounds remained significant as a protective factor (OR = 0.73, 95%CI: 0.57‒0.94). The findings of this study suggest that dietary supplements, particularly ferric compounds, may have implications for managing abdominal obesity. These findings could inform public health strategies by incorporating ferric compound supplementation into dietary interventions aimed at reducing abdominal obesity prevalence. Furthermore, the potential clinical application of ferric compounds highlights the need for integrating nutritional approaches in obesity management programs. However, longitudinal studies are needed to establish causal relationships and confirm the long-term effectiveness and safety of such interventions.

**Supplementary Information:**

The online version contains supplementary material available at 10.1038/s41598-025-85136-6.

## Introduction

Abdominal obesity, commonly referred to as central or visceral obesity, indicates an augmentation in the accumulation of fat within the abdominal area^1^. This particular type of fat possesses a high metabolic activity and consistently releases free fatty acids (FFA) into the portal circulation^2^. The release of these fatty acids, coupled with some inflammatory cytokines production like tumor necrosis factor-alpha (TNF-α) and interleukin-6 (IL-6), instigates systemic inflammation and the onset of metabolic disorders like cardiovascular disease and type 2 diabetes^3–5^. Furthermore, the escalation of inflammatory cytokines creates a favorable environment for the emergence of other ailments such as depression, kidney diseases, psoriasis, and various cancers, including esophageal, stomach, and lung cancers^3,6,7^. Meanwhile, reflux esophagitis^8^ and hip fracture^9^ have been positively connected with abdominal obesity. Moreover, emerging evidence suggests a strong association between abdominal obesity and elevated levels of specific adipokines which further exacerbate metabolic dysfunction^10^. Given these circumstances, it is imperative to prioritize the management of abdominal obesity in order to enhance the well-being of affected individuals and reduce its prevalence.

The utilization of vitamin and mineral supplements has long been considered a viable approach for disease prevention and treatment. Presently, there has been a surge in the popularity of these supplements, with some being recommended to promote overall health and resistance^11^. In the United Kingdom, it is estimated that over 50% of adults consume at least one dietary supplement daily^12^. Iran has also witnessed success in supplement usage, with up to a quarter of individuals in certain cohort studies reporting their utilization^13^. Many individuals incorporate nutritional supplements into their routines, believing that these compounds can alleviate their disease symptoms and contribute significantly to their overall health restoration^14^. This belief has been the subject of numerous studies, yielding valid but different and occasionally conflicting results. Some studies have demonstrated positive effects of certain supplements, such as vitamin D, in preventing acute and chronic diseases. Vitamin D plays a crucial role in the regulation and maintenance of innate immunity^15^. Research findings indicate that vitamin D deficiency is associated with increased incidence of both bacterial and viral acute respiratory diseases, as well as non-communicable chronic diseases such as obesity, type 2 diabetes and metabolic syndrome^16^. However, the majority of studies failed to provide sufficient evidence for the beneficial effects of supplements on cognitive function, verbal memory, cardiovascular diseases, cancer, or mortality^17–20^. Studies investigating the impact of supplements on abdominal obesity have yielded inconsistent results, while some have highlighted the existence of a relationship^21^, others have pointed to the absence of one^22^.

The mechanisms underlying the association between abdominal obesity and supplement use have not been well understood; however, there are some propositions about potential mechanisms through which supplement intake might influence abdominal obesity or fat distribution. For instance, calcium has been shown to enhance fat oxidation and promote thermogenesis by increasing the activity of uncoupling proteins, leading to decrease weight gain and limit fat accumulation within fat cells^23^. Moreover, dietary calcium may help reduce appetite^24^ and facilitate fat elimination through feces^25^, which could play a role in preventing weight gain.

There is a scarcity of research pertaining to the association between the utilization of supplements and the presence of abdominal obesity in Iran. Consequently, the aim of this investigation was to explore, for the first time, the association between the consumption of various categories of supplements and abdominal obesity in a large Iranian cohort named “Shahedieh Yazd cohort study”.

## Materials and methods

### Design and population

**Fig. 1:**
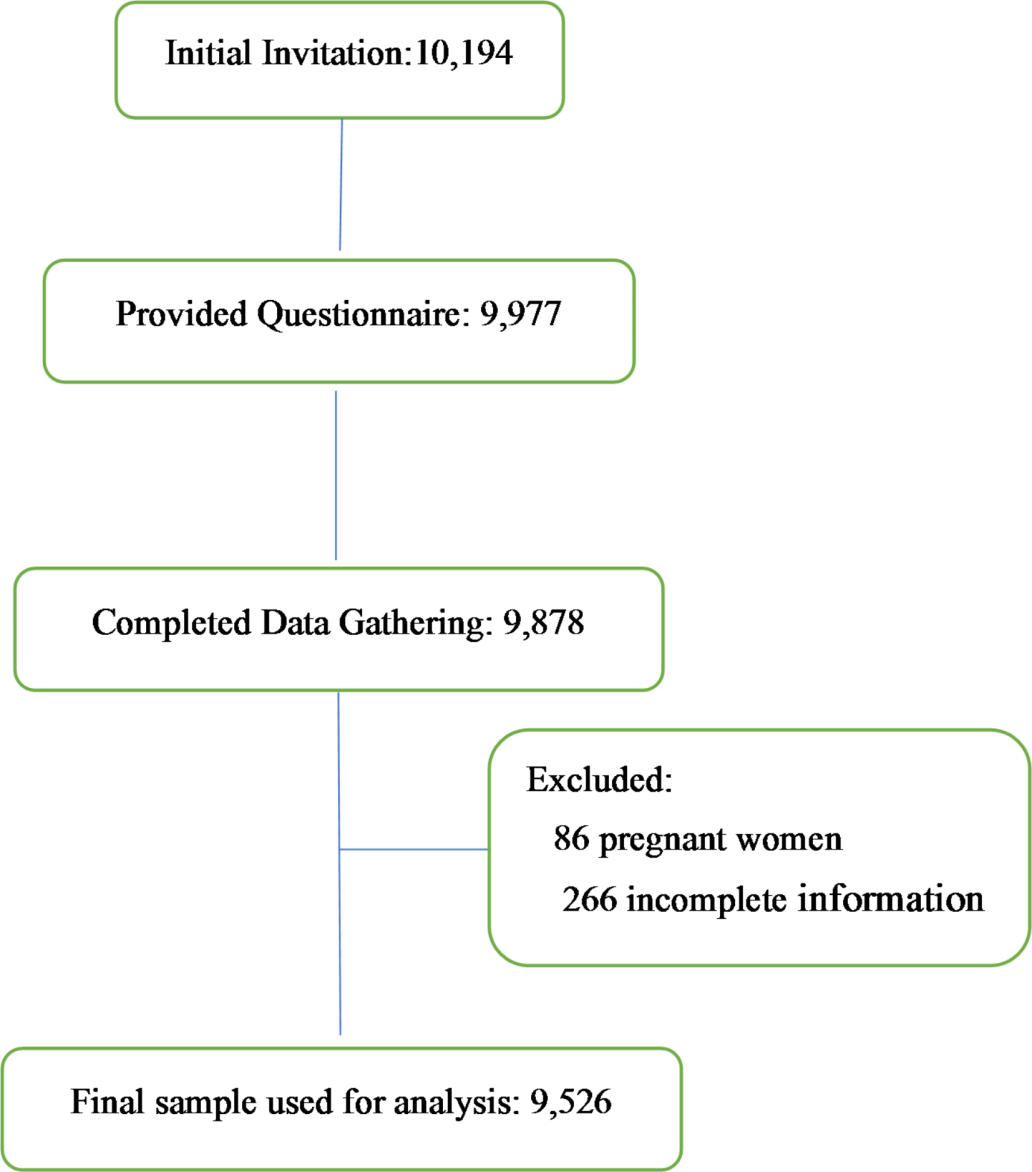
A flow chart illustrating the participant selection process from the Shahedieh cohort study.

The current investigation was an analytical cross-sectional one that was performed on the initial phase data of the Shahedieh cohort study^26^, a population-based and a part of the Persian prospective cohort study^27^. The purpose of this project was to investigate non-communicable diseases and their risk factors in the population aged 35 to 70 years, and it was conducted in the period of May 2015 to October 2017 in the three adjacent cities of Shahedieh, Zarch and Ashkezar, located in Yazd province, located at the center of Iran. The reasons for choosing this area were various factors such as accessibility, low immigration and the possibility of attracting the cooperation of local people. Inclusion criteria included living in mentioned cities for more than 9 months within a year and Iranian nationality. People who physically or psychologically were unable to complete the study enrolment process were excluded. In the first phase of the cohort study, 10,194 people were invited to participate, of which 9977 people were provided with a questionnaire and 9878 completed the data gathering process. For conducting the present study, 86 women due to pregnancy and 266 individuals due to incomplete information about their waist circumference or the use of supplements were excluded, and finally the data of 9526 people were utilized (Fig. 1). Further information regarding the protocol of the PERSIAN cohort study is available in other sources (27).

## Sample size calculation

The sample size needed to achieve the main goal of present study was calculated based on the prevalence of abdominal obesity and supplement use in Iran. According to the literature, the prevalence of abdominal obesity was reported to be 41.8%^28^ while the prevalence of supplement use was 24.7% in the Iranian population^13^. The sample size was calculated as 1754 using the formula below, with α = 0.05, *P* = 24.7, and d = 0.02.


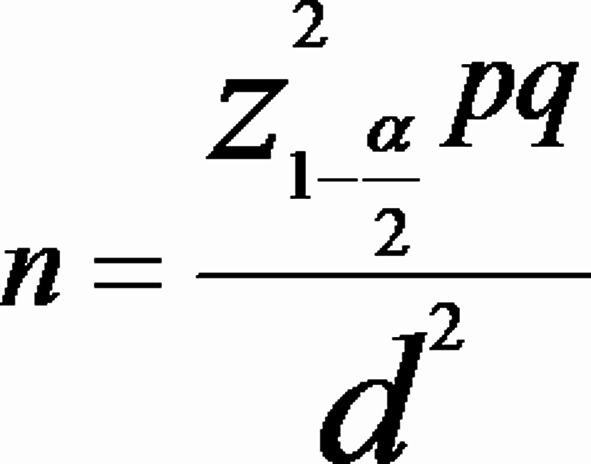


Since the least sample size we needed was lower than the whole number of participants of the Shahedieh cohort study, therefore we included the data of all participants in the analysis process.

### Data collection

The data used for performing present research was derived from the initial phase data of the Shahedieh cohort study, as mentioned before. In that study, people’s data was collected in person and through face-to-face interviews conducted by trained inquirers. Once the informed consent was obtained from the participants, demographic characteristics and type of consumed nutritional supplements as well as lifestyle information such as diet, physical activity and smoking were evaluated through valid questionnaires. In addition, anthropometric indicators, including height, weight, and waist circumference, were measured with high accuracy by professional individuals, so that the weight without shoes and with minimal covering was measured by a nutritionist with the help of a digital scale with 100-gram accuracy. In addition, height was measured with the help of a tape measure attached to the wall, while the people were in a standing position next to the wall without shoes and were completely attached to the wall behind the head, with an accuracy of one centimeter. Also, the waist circumference in the narrowest part of the body was measured in a position where the person was at the end of his natural exhalation, with the help of an inflexible tape measure without putting pressure on the body^27^.

## Calculations

The use of any of the supplements or the use of at least one of them was considered positive if it was being used at the time of completing the questionnaire or had been employed within the preceding 12 months. The level of physical activity was calculated based on the International Physical Activity Questionnaire (IPAQ) and with the help of the total MET score. Physical activity was then divided into three categories: low (less than 30 min per week), moderate (30 to 120 min per week), and high physical activity (more than 120 min per week). Body mass index (BMI) was calculated based on the relevant formula and by dividing weight in kilograms by height in meters with hundredths of a decimal. Then, based on the obtained number, BMI less than 25 was considered as normal, 25-29.9 as overweight, and 30 and more as general obesity. In addition, a waist circumference higher than 88 cm in women and higher than 102 cm in men was considered as abdominal obesity^29^.

### Data analysis

Statistical analysis was performed using the SPSS version 22 software. Descriptive statistics were reported for quantitative variables as the mean ± standard deviation, and for qualitative variables as frequency (percentage). The association between abdominal obesity and each supplement was investigated using the Chi-square test. Simple logistic regression was utilized to examine the association between each supplement (either as a risk factor or protective factor) as independent variables and abdominal obesity as dependent variable. Initially, the variables were entered one by one into the model and the simple logistic regression test was conducted. Subsequently, the variables with a *P*-value less than 0.2 were included in the Backward Multivariable logistic regression model (Backward Stepwise Regression) to identify the significant variables.

Backward Stepwise Regression is an iterative procedure that starts with a full model containing all variables under consideration. At each step of the analysis, the least significant variables are eliminated from the model. This process continues until a reduced model is obtained that provides the best explanation for the data, or until no variables remain in the model.

To achieve the objectives of the present study, all significant demographic and supplement variables including age, gender, education, BMI, smoking, physical activity, multivitamin, calcium, vitamin D, folic acid, Omega 3, ferric compounds and other supplements were initially included in the analysis (initial multivariable analysis). Subsequently, the supplement variables were removed from the model and replaced with the variable “use of at least one supplement” (second multivariable analysis).

No imputation for missing data was applied as participants with incomplete data were excluded from the analysis. The results were expressed as odds ratios with 95% confidence intervals. A *P*-value of less than 0.05 was considered statistically significant.

### Ethical considerations

In the first phase of Shahedieh cohort study, ethical considerations were fully observed in which the study process was thoroughly described and then informed consent was completed by either the participants or their authorized legal representative (if they were illiterate), prior to the inclusion in the study. Since the present project data was a part of Shahedieh cohort study dataset and also the data was extracted without confidential information, there was no need to get informed consent from the individuals once more, according to country’s regulations.

This study was conducted based on the instructions contained in the Declaration of Helsinki and all related procedures were approved by the Ethics Committee of Shahid Sadoughi University of Medical Sciences in Yazd with the code of ethics approval IR.SSU.MEDICINE.REC.1399.288.

## Results

In total, 9458 participants with average age of 47.4 ± 9.6 years were involved in this study. Among them, 4783 were men with an average age of 48.2 ± 9.6 years and 4675 were women with an average age of 47.2 ± 9.5 years. Out of the entire sample, 4785 individuals, which represents 50.6% of the participants, presented abdominal obesity, and 4093 individuals, accounting for 43.3% of the sample, reported the use of at least one type of supplements.

The most commonly used supplement was vitamin D with 2256 consumers (23.9%), followed by calcium (1975 people ‒ 20.9%), ferric compounds (1559 people ‒ 16.5%), folic acid (1143 people ‒12.1%), other supplements (1095 people ‒ 11.6%), omega-3 (767 people ‒ 8.1%) and multivitamins (574 people ‒ 5.8%). Zinc, on the other hand, was the least utilized supplement, with only 102 individuals (1.1%) reporting its usage.

Female gender, no smoking, older age, higher body mass index, lower literacy, and physical inactivity all exhibited significant positive associations with abdominal obesity (P value < 0.001). Additionally, the consumption of all supplements, except for zinc, was significantly related to abdominal obesity (P value < 0.001; except for Multi-vitamin P value = 0.049) (Table 1).

**Table 1:** Prevalence of demographic factors and supplement use in terms of abdominal obesity in people aged 35 to 70 years old, Shahedieh Cohort Study.

Variable	Category	Abdominal obesity		*P* value
		Yes	No	
Gender, N (%)	Male	1111 (23.2%)	3672 (78.6%)	< 0.001
	Female	3674 (76.8%)	1001 (21.4%)	
Age (Yr), Mean (SD)	‒	48.62 ± 9.58	46.78 ± 9.54	< 0.001
Education, N (%)	Illiterate	1098 (23)	471 (10)	< 0.001
	Under diploma	2477 (51.7)	2070 (44)	
	Diploma	730 (15.2)	1163 (25)	
	University	480 (10)	969 (21)	
BMI, N (%)	Normal	196 (4.1)	2066 (44.2)	< 0.001
	Overweight	1688 (35)	2326 (49.7)	
	Obese	2900 (60.9)	281 (6.1)	
Smoking, N (%)	Yes	521 (11)	1592 (35)	< 0.001
	No	4203 (89)	2995 (65)	
Physical activity, N (%)	Low	2798 (58.5)	2062 (44)	< 0.001
	Intermediate	1688 (35.5)	1858 (40)	
	High	299 (6)	753 (16)	
Multi-vitamin, N (%)	Yes	299 (6)	248 (5)	0.049
	No	4486 (94)	4425 (95)	
Calcium, N (%)	Yes	1319 (28)	656 (14)	< 0.001
	No	3466 (72)	4017 (86)	
Vitamin D	Yes	1483 (31)	773 (17)	< 0.001
	No	3302 (69)	3900 (83)	
Folic acid	Yes	794 (17)	349 (7)	< 0.001
	No	3991 (83)	4324 (93)	
Omega 3	Yes	498 (10)	269 (6)	< 0.001
	No	4287 (90)	4404 (94)	
Ferric compounds	Yes	1075 (22)	484 (10)	< 0.001
	No	3710 (88)	4189 (90)	
Zinc	Yes	54 (1)	48 (1)	0.633
	No	4731 (99)	4625 (99)	
Other supplements	Yes	639 (13)	456 (10)	< 0.001
	No	4146 (87)	4217 (90)	
Any supplement	Yes	2525 (53)	1568 (34)	< 0.001
	No	2260 (47)	3105 (66)	

The results of the simple logistic regression analysis indicated that advanced age (OR:1.02, 95%CI:1.01–1.03), female gender (OR: 12.13, 95%CI:11.01–13.36), body mass index (BMI) exceeding 25 (overweight OR:7.65, 95%CI:6.52–8.97; obesity OR: 108.78, 95%CI:89.88-131.67), and the consumption of all investigated food supplements except zinc (Multivitamin OR: 1.19, 95%CI:0.99–1.41; Calcium OR: 2.33, 95%CI: 2.10–2.58; Vitamin D OR: 2.26, 95%CI: 2.05–2.50; Folic acid OR: 2.46, 95%CI: 2.15–2.81; Omega-3 OR: 1.90, 95%CI: 1.63–2.22; Ferric compounds OR: 2.51, 95%CI: 2.23–2.82; Other supplements OR: 1.42, 95%CI:1.25–1.62; Any supplement OR: 2.21, 95%CI:2.02–2.40) were identified as risk factors for abdominal obesity. Conversely, engaging in moderate and high levels of physical activity (in comparison to low physical activity), smoking, and possessing an education level (below a diploma, a diploma, or a university degree compared to illiteracy) were found to be protective factors against abdominal obesity (Table 2).

**Table 2:** Simple logistic regression analysis with the presence of abdominal obesity variable (dependent variable) and demographic, background and supplement use variables (independent variables).

Variable	Category	B coefficient	Crude OR (95%CI)	*P*-value
Age (Yr)	‒	0.020	1.02 (1.01–1.03)	< 0.001
Gender (F/M)	‒	2.496	12.13 (11.01–13.36)	< 0.001
Physical activity	Low	‒	Ref	‒
	Moderate	-0.401	0.67 (0.61–0.73)	< 0.001
	High	-1.229	0.29 (0.25–0.34)	< 0.001
Smoking (Yes)	‒	1.456	0.23 (0.21–0.26)	< 0.001
Education	Illiterate	‒	Ref	‒
	Under diploma	-0.667	0.51 (0.45–0.58)	< 0.001
	Diploma	-1.312	0.27 (0.23–0.31)	< 0.001
	University	-1.549	0.21 (0.18–0.25)	< 0.001
BMI	< 25	‒	Ref	‒
	25-29.9	2.035	7.65 (6.52–8.97)	< 0.001
	≥ 30	4.689	108.78 (89.88-131.67)	< 0.001
Multivitamin (Yes)	‒	0.173	1.19 (0.99–1.41)	0.051
Calcium (Yes)	‒	0.846	2.33 (2.10–2.58)	< 0.001
Vitamin D (Yes)	‒	-0.818	2.26 (2.05–2.50)	< 0.001
Folic acid (Yes)	‒	0.902	2.46 (2.15–2.81)	< 0.001
Omega-3 (Yes)	‒	0.643	1.90 (1.63–2.22)	< 0.001
Ferric compounds (Yes)	‒	0.919	2.51 (2.23–2.82)	< 0.001
Zinc (Yes)	‒	0.095	1.10 (0.74–1.63)	0.633
Other supplements (Yes)	‒	0.354	1.42 (1.25–1.62)	< 0.001
Any supplement (Yes)	‒	0.794	2.21 (2.03–2.40)	< 0.001

In the initial multivariable analysis, the variables of age, gender, smoking, BMI, and the consumption of folic acid and ferric compounds supplements were retained. Based on the findings of the Backward Stepwise Regression test, the likelihood of developing abdominal obesity increased by 5% for each year of age. Furthermore, women were revealed to be 40.6 times more susceptible to abdominal obesity than men (95%CI: 33.37‒49.43; P value < 0.001). Individuals who were classified as overweight had a 13.8 times higher risk of experiencing abdominal obesity than those with a normal weight (OR:13.83, 95%CI: 11.38‒16.79; P value < 0.001). Additionally, general obesity remained the strongest risk factor for this condition, elevating the likelihood of abdominal obesity by 389 times (OR:388.09, 95%CI: 300.31‒501.54; P value < 0.001). It is worth noting that after controlling for the remaining variables in the final model, smoking no longer exhibited an association with abdominal obesity.

Among the remaining supplements included in the final model, namely ferric compounds and folic acid, only ferric compounds demonstrated a significant association with abdominal obesity and was recognized as a protective factor (OR:0.73, 95%CI: 0.57–0.94; P value = 0.016). Specifically, the likelihood of developing abdominal obesity in individuals who consumed ferric compounds was 27% lower compared to those who did not use this supplement (Table 3). In the second multivariable analysis, the variables of age, gender, smoking, and BMI were retained, while the variable “use of at least one supplement” was excluded from the model (Table 4).

**Table 3:** Backward stepwise regression analysis with the presence of abdominal obesity (dependent variable) and demographic, background and supplement variables (independent variables).

Variable	Category	B coefficient	Adjusted OR (95%CI)	*P*-value
Age (Yr)	‒	0.051	1.05 (1.04‒1.06)	< 0.001
Gender (F/M)	‒	3.704	40.61 (33.37‒49.43)	< 0.001
Smoking, Yes	‒	0.168	1.18 (0.97‒1.43)	0.082
BMI	< 25	‒	Ref	
	25-29.9	2.627	13.83 (11.38‒16.79)	< 0.001
	≥ 30	5.961	388.09 (300.31‒501.54)	< 0.001
Folic acid, Yes	‒	0.269	1.30 (0.99‒1.71)	0.053
Ferric compounds, Yes	‒	-0.305	0.73 (0.57‒0.94)	0.016

**Table 4:** Backward stepwise regression analysis with the presence of abdominal obesity (dependent variable) and demographic, background and “any supplement use” variables (independent variables).

Variable	Category	B coefficient	Adjusted OR (95%CI)	*P*-value
Age (Yr)	‒	0.052	1.05 (1.04‒1.06)	< 0.001
Gender (F/M)	‒	3.666	39.08 (32.42‒47.12)	< 0.001
Smoking, Yes	‒	0.167	1.18 (0.97‒1.43)	0.085
BMI	< 25	‒	Ref	
	25-29.9	2.619	13.72 (11.30‒16.65)	< 0.001
	≥ 30	5.956	386.11 (298.92‒498.74)	< 0.001

## Discussion

In the current investigation, subsequent to conducting Backward stepwise regression analysis, the variables of age, gender, BMI, smoking, and two supplements comprising ferric compounds and folic acid persisted in the model. Between these two supplements, solely ferric compounds exhibited a significant association with abdominal obesity and was acknowledged as a protective factor, leading to a 27% lower likelihood of abdominal obesity in individuals who consumed ferric compounds compared to those who did not utilize this supplement.

The findings of the studies demonstrated that ferric compounds deficiency anemia can result in fatigue and further exacerbate weight gain by diminishing physical activity^30^. Additionally, observations from mouse model investigations revealed that ferric compounds supplementation reduces weight gain induced by diet and intrahepatic fat accumulation. It appears that ferric compounds supplementation mitigates the morphological irregularities of mitochondria in skeletal muscle and enhances the expression of genes linked to the mitochondrial electron transport chain and energy metabolism in skeletal muscle and liver^31^. Consequently, it is plausible that ferric compounds supplementation can yield a favorable impact on reducing abdominal circumference and ameliorating central obesity.

In the present study, the association between calcium supplementation and abdominal obesity was found to be insignificant. Earlier research has indicated an inverse correlation between calcium intake through diet and abdominal obesity, indicating that a decrease in calcium intake is associated with an increase in abdominal fat^32^. Conversely, an increase in calcium intake through diet diminishes the risk of abdominal obesity^22^. Furthermore, the outcomes of a meta-analysis revealed that the consumption of low-fat dairy products, as part of a diet with restricted energy intake, contributes to body fat loss over a period of 4 months, whereas calcium supplements do not yield the same effect^33^.

Contrary to expectations, the results of studies pertaining to supplemental calcium present a different picture. According to the obtained results, calcium obtained through supplements rather than through diet has minimal or no impact on weight or body composition^33^. For instance, the study conducted by Huang et al. on 8940 adults aged 20 to 74 demonstrated that while dietary calcium is associated with a decrease in abdominal obesity in women, supplemental calcium consumption has no relationship with abdominal obesity and body composition in either the male or female groups^22^.

The proposition has been put forward that dairy sources of calcium have a notable impact on weight and fat gain reduction, as well as an acceleration of fat loss, to a greater extent than calcium supplements. This augmented effect of dairy products relative to supplemental calcium is likely attributed to additional bioactive compounds, such as angiotensin-converting enzyme inhibitors, and the abundant concentration of branched-chain amino acids, which work synergistically with calcium to diminish obesity^34^.

Within present investigation, no significant association between vitamin D supplementation and abdominal obesity was discovered. As documented in certain sections of the literature, vitamin D supplementation does not exert a substantial influence on abdominal obesity. Specifically, a meta-analysis conducted in 2020 encompassing 11 clinical trials exploring the association between vitamin D and abdominal obesity indicated that vitamin D did not significantly decrease waist circumference when compared to a placebo^35^. Furthermore, another meta-analysis conducted in 2023 concerning the association between vitamin D and abdominal obesity in patients with metabolic syndrome concluded that vitamin D supplementation induced a significant reduction in waist circumference in only one out of five studies. The researchers conducting the study emphasized that the low certainty of evidence did not support the hypothesis of waist circumference reduction due to vitamin D supplementation^36^. Nevertheless, apart from these studies, a meta-analysis concluded that there was an inverse relationship between vitamin D blood levels and abdominal circumference, whereby every 25 nmol/liter increase in vitamin D levels increased the risk of abdominal obesity by 10%^37^. Most of the active vitamin D is supplied in the body via exposure to sunlight, while the contribution from dietary intake is negligible. However, supplements can alleviate vitamin D deficiency. Research findings indicate that the bioavailability of various types of vitamin D supplements differs, resulting in varying levels of vitamin D in the blood^38^. However, once vitamin D enters the bloodstream and attains an optimal level, it can mitigate the risk of abdominal obesity^37^. Thus, one possible explanation for the lack of association between vitamin D supplement usage and abdominal obesity in some parts of the research like the current study may be the failure to attain the optimal blood level of vitamin D.

In the present study, there was no correlation between omega-3 consumption and abdominal obesity. However, a meta-analysis of 11 RCTs (updated to 2015) concluded that omega-3 PUFA may effectively reduce waist circumference and triglyceride levels in overweight and obese adults, although it may not effectively reduce body weight. Of course, it was emphasized in this study that due to the small number and poor quality of RCTs in the meta-analysis, the results obtained were not definitive^39^. In addition, in a clinical trial study that was conducted in 2017 to determine the effect of omega-3 on weight reduction in obese people on a weight loss diet, two groups including the weight loss diet group and the weight loss diet group plus omega 3 were included^21^. In this study, a significant decrease in weight, waist circumference, and BMI was observed in both groups, but the mass and percentage of abdominal fat in the omega-3 group decreased significantly more than the control group. According to the findings of this study, omega-3 PUFA supplementation decreased the mass and percentage of abdominal fat in overweight or obese people on a weight loss diet. Therefore, it can be concluded that omega-3 consumption can play an effective role in reducing abdominal circumference. The bottom line, what draws attention in these studies is that the dose of omega-3 used in the mentioned studies was between 1.2 and 5 g per day^40^. The reason for this could be that in Iran, during the time of the study, the omega-3 products were primarily consumed with the intention of prevention, resulting in a dosage of less than 1000 mg per day. Hence, it appears that one of the rationales for the absence of mentioned association in the current investigation may be the substantially lower dosage administered to the study participants compared to the dosage employed in the aforementioned studies.

In this investigation, folic acid exhibited a noteworthy association with abdominal obesity in the univariable logistic regression analysis; however, this association failed to retain its significance in the multivariable analysis. The investigation of folic acid’s role as a supplement in abdominal obesity has been conducted in animal and human studies, resulting in conflicting findings. For example, a 2016 study on mice by Kelly et al. demonstrated that consuming a high-fat diet with extra folic acid may cause weight gain, heightened fat mass, adipose tissue inflammation, and systemic glucose intolerance^41^. However, a case-control study was carried out by Mlodzik-Czyzewska et al. in 2020, focusing on healthy, obese, and non-obese individuals, revealed an association between a reduced intake of folate and its low serum levels with both higher body mass index and increased fat accumulation in the abdominal region^42^. Moreover, a systematic review-meta-analysis conducted in 2018 demonstrated no correlation between blood folate levels and body mass index among individuals^43^. Given the contradicting findings concerning folate intake and its serum levels, and abdominal obesity, it is imperative to conduct further investigations in this particular field.

In current research, we defined abdominal obesity using waist circumference cutoffs of 102 cm for men and 88 cm for women, which are commonly applied in clinical and research settings. However, alternative cutoffs exist depending on specific population characteristics, such as ethnicity and health risk profiles. Using a lower cutoff would likely increase the prevalence of abdominal obesity in our sample, potentially strengthening the observed associations with supplement use, whereas higher thresholds might reduce the prevalence and weaken these associations. Since the relationships identified in this study could be influenced by the choice of cutoff values, selecting a more common standard definition might guarantee comparability across studies.

This study provides a unique contribution to the literature by highlighting a specific association between the use of ferric compounds and a reduced risk of abdominal obesity. While previous research has largely focused on the general health benefits of iron^44^, few studies have explored the role of iron supplementation in reducing central adiposity specifically. Our findings suggest a potential protective effect of ferric compounds on abdominal obesity, which could be a novel area for further investigation and may differentiate this study from others in the field. Moreover, this research is the first large-scale investigation within an Iranian population to examine the relationship between dietary supplement use and abdominal obesity. Given the specific dietary habits and genetic factors of this population, our study not only adds to the understanding of these associations within Iran but also provides a foundation for comparative studies in other populations.

The findings of this study highlight the role of dietary supplements, particularly ferric compounds, in abdominal obesity. However, abdominal obesity is influenced by a multifaceted interplay of demographic, behavioral, and biological factors. Our results suggest that supplement use may interact with variables such as gender, BMI, and some other lifestyle factors, potentially modifying their impact on abdominal obesity. For instance, the observed gender differences in supplement consumption patterns could reflect underlying disparities in health behaviors or metabolic responses. Similarly, the relationship between BMI and abdominal obesity might be further modulated by the type and frequency of supplement use. Future studies are needed to explore these complex interactions and their implications for public health interventions.

### Implications for practice and future research

The findings from this study suggest that dietary supplements, particularly ferric compounds, may have implications for managing abdominal obesity. In clinical practice, healthcare providers might consider the potential role of ferric supplementation as part of a broader nutritional and lifestyle intervention strategy for individuals at risk of abdominal obesity. However, these results should be interpreted with caution, as they are based on a cross-sectional study. Therefore, longitudinal studies are needed to establish causal relationships. Future research should focus on exploring the underlying biological mechanisms that may link ferric compounds with central adiposity, such as their effects on inflammation and lipid metabolism. Additionally, it would be valuable to investigate whether similar patterns are observed in different populations with varying dietary and genetic backgrounds to assess the generalizability of these findings. Further randomized controlled trials examining the effects of iron and other specific supplements on abdominal obesity could provide more robust evidence for clinical guidelines and recommendations.

### Strengths and limitations

The current study was conducted on the data derived from a large comprehensive population of Iranian 35–70 year‒adults using valid instruments and professional interviewers. Despite these strengths, there were some limitations in our investigation. At first, this study utilized a cross-sectional design, which inherently limits the ability to establish causal relationships between supplement consumption and abdominal obesity. While our findings provide valuable insights into potential associations, they should be interpreted with caution. Longitudinal studies are required to confirm causality and further investigate the temporal dynamics of these relationships. Second, in this study, waist circumference was used as the sole measure of abdominal obesity, with cutoff values based on international standards. These thresholds were selected to facilitate comparisons of our findings with studies conducted in other countries. However, using non-Iranian cutoff values may not fully reflect the specific anthropometric characteristics of the Iranian population, which could impact the accuracy of obesity classification. Third, the complete and accurate data on dosage and duration and also being present or past users of the supplements was not accessible for the researchers. Forth, the exact contents of some supplements such as multivitamins were not specified. Fifth, despite the large sample size, the participants in this study were not representative of the entire population of Iran because this population belongs to a limited region within one of the cities located in Yazd province, which is one of the thirty-one provinces of Iran.

## Conclusion and future perspectives

Abdominal obesity is a complex condition that is influenced by a variety of factors. The results of this study highlight the potential benefits of increased ferric compounds supplementation in reducing the risk of abdominal obesity. However, given the finding of present study on one hand and above-mentioned limitations on the other hand, it is recommended that comprehensive randomized clinical trials be designed to evaluate the precise dosage and duration of supplementation on abdominal obesity. Furthermore, future studies should consider using more accurate tests, such as body composition analysis, to assess abdominal obesity.

## Electronic supplementary material

Below is the link to the electronic supplementary material.

Supplementary Material 1

## Data Availability

The data from the present investigation can be obtained from the corresponding author upon an acceptable and reasonable request.

## References

[1] Misra, A. et al. Consensus statement for diagnosis of obesity, abdominal obesity and the metabolic syndrome for Asian indians and recommendations for physical activity, medical and surgical management. *Japi* **57** (2), 163–170 (2009). 19582986

[2] Chait, A. & Den Hartigh, L. J. Adipose tissue distribution, inflammation and its metabolic consequences, including diabetes and cardiovascular disease. *Front. Cardiovasc. Med.* **7**, 522637 (2020). 10.3389/fcvm.2020.00022 PMC7052117 32158768

[3] Ellulu, M. S., Patimah, I., Khaza’ai, H., Rahmat, A. & Abed, Y. Obesity and inflammation: the linking mechanism and the complications. *Archives Med. Sci.* **13** (4), 851–863 (2017). 10.5114/aoms.2016.58928 PMC5507106 28721154

[4] Rahimlou, M., Mirzaei, K., Keshavarz, S. A. & Hossein-Nezhad, A. Association of circulating adipokines with metabolic dyslipidemia in obese versus non-obese individuals. *Diabetes Metabolic Syndrome: Clin. Res. Reviews*. **10** (1), S60–S5 (2016). 10.1016/j.dsx.2015.09.015 26482964

[5] Morshedzadeh, N. et al. The effects of flaxseed supplementation on metabolic syndrome parameters, insulin resistance and inflammation in ulcerative colitis patients: an open-labeled randomized controlled trial. *Phytother. Res.* **35** (7), 3781–3791 (2021). 33856729 10.1002/ptr.7081

[6] Du, X., Hidayat, K. & Shi, B-M. Abdominal obesity and gastroesophageal cancer risk: systematic review and meta-analysis of prospective studies. *Biosci. Rep.* **37** (3), BSR20160474 (2017). 28336766 10.1042/BSR20160474 PMC5426287

[7] Hidayat, K., Du, X., Chen, G., Shi, M. & Shi, B. Abdominal obesity and lung cancer risk: systematic review and meta-analysis of prospective studies. *Nutrients* **8** (12), 810 (2016). 27983672 10.3390/nu8120810 PMC5188465

[8] Zhan, J. et al. Abdominal obesity increases the risk of reflux esophagitis: a systematic review and meta-analysis. *Scand. J. Gastroenterol.* **57** (2), 131–142 (2022). 34738858 10.1080/00365521.2021.1994643

[9] Sadeghi, O., Saneei, P., Nasiri, M., Larijani, B. & Esmaillzadeh, A. Abdominal obesity and risk of hip fracture: a systematic review and meta-analysis of prospective studies. *Adv. Nutr.* **8** (5), 728–738 (2017). 28916573 10.3945/an.117.015545 PMC5593104

[10] Rahimlou, M., Mirzaei, K., Keshavarz, S. A. & Hossein-Nezhad, A. Association of circulating adipokines with metabolic dyslipidemia in obese versus non-obese individuals. *Diabetes Metabolic Syndrome*. **10** (1 Suppl 1), S60–S65 (2016). 26482964 10.1016/j.dsx.2015.09.015

[11] Djaoudene, O. et al. A global overview of dietary supplements: regulation, market trends, usage during the COVID-19 pandemic, and health effects. *Nutrients* **15** (15), 3320 (2023). 37571258 10.3390/nu15153320 PMC10421343

[12] Henderson, L., Gregory, J. & Swan, G. The National Diet and Nutrition Survey: adults aged 19 to 64 years. Vitamin and mineral intake and urinary analytes. ;**3**:14–37. (2003).

[13] Mahdavi-Roshan, M. et al. Dietary supplements consumption and its association with socioeconomic factors, obesity and main non-communicable chronic diseases in the north of Iran: the PERSIAN Guilan Cohort Study (PGCS). *BMC Nutr.* **7** (1), 84 (2021). 34906216 10.1186/s40795-021-00488-2 PMC8672625

[14] Akilen, R., Tsiami, A. & Robinson, N. Individuals at risk of metabolic syndrome are more likely to use a variety of dietary supplements. *Adv. Integr. Med.* **1** (3), 131–137 (2014).

[15] Martens, P-J., Gysemans, C., Verstuyf, A. & Mathieu, C. Vitamin D’s effect on immune function. *Nutrients* **12** (5), 1248 (2020). 32353972 10.3390/nu12051248 PMC7281985

[16] Álvarez-Mercado, A. I., Mesa, M. D., Gil, Á. & Vitamin, D. Role in chronic and acute diseases. *Encyclopedia Hum. Nutr.* :535. (2023).

[17] Lin, J. S. et al. *Screening for Cognitive Impairment in Older Adults* (an evidence update for the US Preventive Services Task Force [Internet], 2013).

[18] Fortmann, S. P., Burda, B. U., Senger, C. A., Lin, J. S. & Whitlock, E. P. Vitamin and mineral supplements in the primary prevention of cardiovascular disease and cancer: an updated systematic evidence review for the US Preventive Services Task Force. *Ann. Intern. Med.* **159** (12), 824–834 (2013). 24217421 10.7326/0003-4819-159-12-201312170-00729

[19] Grodstein, F. et al. Long-term multivitamin supplementation and cognitive function in men: a randomized trial. *Ann. Intern. Med.* **159** (12), 806–814 (2013). 24490265 10.7326/0003-4819-159-12-201312170-00006 PMC3858850

[20] Huang, H-Y. et al. The efficacy and safety of multivitamin and mineral supplement use to prevent cancer and chronic disease in adults: a systematic review for a National Institutes of Health state-of-the-science conference. Annals of internal medicine. ;145(5):372 – 85. (2006). 10.7326/0003-4819-145-5-200609050-00135 16880453

[21] Salman, H. B., Salman, M. A. & Akal, E. Y. The effect of omega-3 fatty acid supplementation on weight loss and cognitive function in overweight or obese individuals on weight-loss diet. *Nutr. Hosp.* **39** (4), 803–813 (2022). 35815739 10.20960/nh.03992

[22] Huang, L. et al. Dietary calcium but not elemental calcium from supplements is associated with body composition and obesity in Chinese women. *PloS One*. **6** (12), e27703 (2011). 22163269 10.1371/journal.pone.0027703 PMC3233543

[23] Rosenblum, J. L., Castro, V. M., Moore, C. E. & Kaplan, L. M. Calcium and vitamin D supplementation is associated with decreased abdominal visceral adipose tissue in overweight and obese adults. *Am. J. Clin. Nutr.* **95** (1), 101–108 (2012). 22170363 10.3945/ajcn.111.019489 PMC3238453

[24] Kabrnová-Hlavatá, K. et al. Calcium intake and the outcome of short-term weight management. *Physiol. Res.* **57** (2), 237–245 (2008). 17552880 10.33549/physiolres.931057

[25] Christensen, R. et al. Effect of calcium from dairy and dietary supplements on faecal fat excretion: a meta-analysis of randomized controlled trials. *Obes. Reviews: Official J. Int. Association Study Obes.* **10** (4), 475–486 (2009). 10.1111/j.1467-789X.2009.00599.x 19493303

[26] Sadeghi, O. et al. Dietary insulin index and dietary insulin load in relation to metabolic syndrome: the Shahedieh Cohort Study. *J. Acad. Nutr. Dietetics*. **120** (10), 1672–1686 (2020). e4. 10.1016/j.jand.2020.03.008 32414656

[27] Poustchi, H. et al. Prospective Epidemiological Research Studies in Iran (the PERSIAN Cohort Study): Rationale, objectives, and design. *Am. J. Epidemiol.* **187** (4), 647–655 (2018). 29145581 10.1093/aje/kwx314 PMC6279089

[28] Efazati, N. et al. General and abdominal obesity trends in the Iranian adult population from 2004 to 2021. *J. Diabetes Metab. Disord.* **22** (2), 1745–1761 (2023). 37975121 10.1007/s40200-023-01310-5 PMC10638213

[29] Grundy, S. M. et al. Diagnosis and management of the metabolic syndrome: an American Heart Association/National Heart, Lung, and Blood Institute scientific statement. *Circulation* **112** (17), 2735–2752 (2005). 16157765 10.1161/CIRCULATIONAHA.105.169404

[30] Muñoz, M., Botella-Romero, F., Gómez-Ramírez, S., Campos, A. & García-Erce, J. Iron deficiency and anaemia in bariatric surgical patients: causes, diagnosis and proper management. *Nutr. Hosp.* **24** (6), 640–654 (2009). 20049366

[31] Kitamura, N. et al. Iron supplementation regulates the progression of high fat diet induced obesity and hepatic steatosis via mitochondrial signaling pathways. *Sci. Rep.* **11** (1), 10753 (2021). 34031430 10.1038/s41598-021-89673-8 PMC8144192

[32] Suhett, L. G. et al. Inverse association of calcium intake with abdominal adiposity and C-reactive protein in Brazilian children. *Public Health. Nutr.* **21** (10), 1912–1920 (2018). 29506595 10.1017/S136898001800023X PMC10260829

[33] Booth, A. O., Huggins, C. E., Wattanapenpaiboon, N. & Nowson, C. A. Effect of increasing dietary calcium through supplements and dairy food on body weight and body composition: a meta-analysis of randomised controlled trials. *Br. J. Nutr.* **114** (7), 1013–1025 (2015). 26234296 10.1017/S0007114515001518

[34] Zemel, M. B. Role of calcium and dairy products in energy partitioning and weight management. *Am. J. Clin. Nutr.* **79** (5), 907S–12S (2004). 15113738 10.1093/ajcn/79.5.907S

[35] Duan, L. et al. Effects of vitamin D supplementation on general and central obesity: results from 20 randomized controlled trials involving apparently healthy populations. *Annals Nutr. Metabolism*. **76** (3), 153–164 (2020). 10.1159/000507418 32645694

[36] Aquino, S. et al. Effects of vitamin D supplementation on cardiometabolic parameters among patients with metabolic syndrome: a systematic review and GRADE evidence synthesis of randomized controlled trials. *Heliyon* (2023). 10.1016/j.heliyon.2023.e20845 PMC10598496 37885733

[37] Hajhashemy, Z., Shahdadian, F., Ziaei, R. & Saneei, P. Serum vitamin D levels in relation to abdominal obesity: a systematic review and dose–response meta-analysis of epidemiologic studies. *Obes. Rev.* **22** (2), e13134 (2021). 32881271 10.1111/obr.13134

[38] Šimoliūnas, E., Rinkūnaitė, I., Bukelskienė, Ž. & Bukelskienė, V. Bioavailability of different vitamin D oral supplements in laboratory animal model. *Medicina* **55** (6), 265 (2019). 31185696 10.3390/medicina55060265 PMC6631968

[39] Zhang, Y., Liu, W., Zhao, T. & Tian, H. Efficacy of omega-3 polyunsaturated fatty acids supplementation in managing overweight and obesity: a meta-analysis of randomized clinical trials. *J. Nutr. Health Aging*. **21** (2), 187–192 (2017).28112774 10.1007/s12603-016-0755-5

[40] Albracht-Schulte, K. et al. Omega-3 fatty acids in obesity and metabolic syndrome: a mechanistic update. *J. Nutr. Biochem.* **58**, 1–16 (2018). 29621669 10.1016/j.jnutbio.2018.02.012 PMC7561009

[41] Kelly, K. B. et al. Excess folic acid increases lipid storage, weight gain, and adipose tissue inflammation in high fat diet-fed rats. *Nutrients* **8** (10), 594 (2016). 27669293 10.3390/nu8100594 PMC5083982

[42] Mlodzik-Czyzewska, M. A., Malinowska, A. M. & Chmurzynska, A. Low folate intake and serum levels are associated with higher body mass index and abdominal fat accumulation: a case control study. *Nutr. J.* **19**, 1–8 (2020). 32498709 10.1186/s12937-020-00572-6 PMC7273685

[43] Wiebe, N., Field, C. & Tonelli, M. A systematic review of the vitamin B12, folate and homocysteine triad across body mass index. *Obes. Rev.* **19** (11), 1608–1618 (2018). 30074676 10.1111/obr.12724

[44] Charlebois, E. & Pantopoulos, K. Nutritional aspects of Iron in Health and Disease. *Nutrients* ;**15**(11). (2023). 10.3390/nu15112441 PMC10254751 37299408

