# Supplementary material for: Association between abdominal obesity and nutritional supplement use among Iranian adults in the Shahedieh cohort study
Source: Sci Rep. 2025 Jan 7;15:1046. doi: 10.1038/s41598-025-85136-6 (PMC11707142; doi:10.1038/s41598-025-85136-6)
Supplement: Supplementary file 1 — Supplementary Material 1 [file 41598_2025_85136_MOESM1_ESM.docx]

**Non-significant findings of each Table**

*Table 1. Prevalence of demographic factors and supplement use in terms of abdominal obesity in people aged 35 to 70 years old, Shahedieh Cohort Study*

| **Variable** | **Category** | **Abdominal obesity** | | **P value** |
| --- | --- | --- | --- | --- |
|  |  | **Yes** | **No** |  |
| Zinc | Yes | 54 (1) | 48 (1) | 0.633 |
|  | No | 4731 (99) | 4625 (99) |  |

*Table 2. Simple Logistic regression analysis with the presence of abdominal obesity variable (dependent variable) and demographic, background and supplement use variables (independent variables)*

| **Variable** | **Category** | **B coefficient** | **Crude OR (95%CI)** | ***P*-value** |
| --- | --- | --- | --- | --- |
| Multivitamin (Yes) | ‒ | 0.173 | 1.19 (0.99-1.41) | 0.051 |
| Zinc (Yes) | ‒ | 0.095 | 1.10 (0.74-1.63) | 0.633 |

*Table 3. Backward Stepwise Regression analysis with the presence of abdominal obesity (dependent variable) and demographic, background and supplement variables (independent variables)*

| **Variable** | **Category** | **B coefficient** | **Adjusted OR (95%CI)** | ***P*-value** |
| --- | --- | --- | --- | --- |
| Smoking, Yes | ‒ | 0.168 | 1.18 (0.97‒1.43) | 0.082 |
| Folic acid, Yes | ‒ | 0.269 | 1.30 (0.99‒1.71) | 0.053 |

*Table 4. Backward Stepwise Regression analysis with the presence of abdominal obesity (dependent variable) and demographic, background and “any supplement use” variables (independent variables)*

| **Variable** | **Category** | **B coefficient** | **Adjusted OR (95%CI)** | ***P*-value** |
| --- | --- | --- | --- | --- |
| Smoking, Yes | ‒ | 0.167 | 1.18 (0.97‒1.43) | 0.085 |
